# Spectral analysis of pair-correlation bandwidth: application to cell biology images

**DOI:** 10.1098/rsos.140494

**Published:** 2015-02-11

**Authors:** Benjamin J. Binder, Matthew J. Simpson

**Affiliations:** 1 School of Mathematical Sciences, University of Adelaide, South Australia, Australia; 2 School of Mathematics, Queensland University of Technology (QUT), Brisbane, Queensland, Australia; 3 Institute of Health and Biomedical Innovation, Queensland University of Technology (QUT), Brisbane, Queensland, Australia

**Keywords:** pair-correlation, spectral analysis, spatial patterns, cell clustering, *in vitro* assay

## Abstract

Images from cell biology experiments often indicate the presence of cell clustering, which can provide insight into the mechanisms driving the collective cell behaviour. Pair-correlation functions provide quantitative information about the presence, or absence, of clustering in a spatial distribution of cells. This is because the pair-correlation function describes the ratio of the abundance of pairs of cells, separated by a particular distance, relative to a randomly distributed reference population. Pair-correlation functions are often presented as a kernel density estimate where the frequency of pairs of objects are grouped using a particular bandwidth (or bin width), Δ>0. The choice of bandwidth has a dramatic impact: choosing Δ too large produces a pair-correlation function that contains insufficient information, whereas choosing Δ too small produces a pair-correlation signal dominated by fluctuations. Presently, there is little guidance available regarding how to make an objective choice of Δ. We present a new technique to choose Δ by analysing the power spectrum of the discrete Fourier transform of the pair-correlation function. Using synthetic simulation data, we confirm that our approach allows us to objectively choose Δ such that the appropriately binned pair-correlation function captures known features in uniform and clustered synthetic images. We also apply our technique to images from two different cell biology assays. The first assay corresponds to an approximately uniform distribution of cells, while the second assay involves a time series of images of a cell population which forms aggregates over time. The appropriately binned pair-correlation function allows us to make quantitative inferences about the average aggregate size, as well as quantifying how the average aggregate size changes with time.

## 2. Introduction

A common feature of images produced during cell biology experiments is the presence of cell clustering. Such clustering is a feature of both *in vitro* [1] and *in vivo* [2] experiments, and can have important consequences. For example, in a clinical setting, melanoma is responsible for most deaths from skin cancer, and early detection of melanoma is essential for successful treatment [3,4]. Many physiological properties associated with melanoma diagnosis (e.g. asymmetry, irregular border, colour irregularity [3,4]) could be associated with cell clustering effects [5]. Alternatively, in an *in vitro* setting, the presence or absence of cell clustering provides important information regarding the mechanisms that govern the rate at which individual cells within the population move and proliferate [1,6–7], as well as providing important information about the strength of cell-to-cell adhesion [8,9]. Given the ubiquitous nature of clustering in cell biology experiments, together with the fact that the degree of clustering is thought to provide insight into relevant biological mechanisms, the development of reliable and informative computational techniques to quantify various properties of the spatial patterns in experimental images is an important task.

Several statistical tools have been developed to make quantitative assessments of the spatial distributions of objects and have been applied to areas such as ecology and natural resource evaluation [10,11]. In this work, we focus on the application of pair-correlation functions, *f*(*δ*) [10–13]. Pair-correlation functions provide a non-dimensional measurement of the frequency of pairs of objects, separated by a distance, *δ*>0, relative to a spatially random reference distribution. When *f*(*δ*)≡1, the frequency of pairs of objects, separated by a distance *δ*, corresponds to the frequency of pairs of objects, separated by the same distance, in a random distribution. When *f*(*δ*)>1, the frequency of pairs of objects, separated by a distance *δ*, is greater than the frequency of pairs of objects, separated by the same distance, in a random distribution and hence the distribution is aggregated at this length scale. Conversely, when *f*(*δ*)<1, the frequency of pairs of objects, separated by a distance *δ*, is less than the frequency of pairs of objects, separated by the same distance, in a random distribution and hence the distribution is segregated at this length scale. One advantage of analysing the pair-correlation function, *f*(*δ*), is that it can provide information regarding the size of objects in the image, the size of clusters in the image and the distance between clusters in the image [14]. For biological images containing a large number of objects, it can be convenient to construct a kernel density estimate of the pair-correlation, *F*, by binning, or grouping, the data at different length scales using a particular bandwidth (or bin width), Δ. Our pair-correlation function is designed for analysing distributions of objects on a two-dimensional square lattice. While many previous analyses have used continuous pair-correlation functions [10–13], our approach is motivated by practical considerations we face when analysing images from cell biology experiments. A standard approach to analyse these images is to use automated image processing software to convert the experimental images into a black and white pixel format. This format is naturally associated with a two-dimensional square lattice which is why we also develop our statistical tools using a two-dimensional square lattice framework.

When calculating the pair-correlation function for a particular biological image, the choice of bandwidth, Δ, is critical. Choosing a large value of Δ results in *F* containing insufficient information as the details of the length scales of the spatial patterning in the image are overly smoothed by the choice of bandwidth. Alternatively, choosing a small value of Δ leads to *F* being dominated by fluctuations. This means that it is difficult to distinguish between meaningful features of the pair-correlation signal and noise introduced by the choice of bandwidth. Presently, there is little guidance available in the literature with regard to making an objective choice of Δ beyond simple trial-and-error or other heuristic approaches [13]. Therefore, a key question of interest is the development of objective methods which allow us to make an appropriate choice of Δ based on the features of the image in question. In this work, we seek to develop, describe and apply such a method by employing spectral techniques to identify Δ.

Spectral techniques have been used previously to analyse spatial patterns [15,16]. For example, previous analyses have directly examined the frequency of distances between objects in particular spatial patterns in spectral space. This kind of analysis leads to data in the form of a periodogram [17,18]. A periodogram (or smoothed periodogram) can help identify dominant features present in a particular spatial pattern [15–18]. Our approach is different as we do not directly examine the frequency of distance between objects in spectral space. The key steps in our approach can be summarized in the following way. First, we examine the distribution of distances between objects in the domain by constructing a pair-correlation function. Second, we examine the discrete Fourier transform of the pair-correlation function, which provides a method to filter the fluctuations in the pair-correlation signal in spectral space, allowing us to identify wavenumbers corresponding to physically relevant length scales. After we identify the relevant length scale, we re-compute the pair-correlation function with a bandwidth corresponding to the length scale associated with a dominant wavenumber and we show, by comparison with synthetic data, that this method reduces the subjectivity in constructing and interpreting pair-correlation signals for spatial patterns.

**Figure 1.**
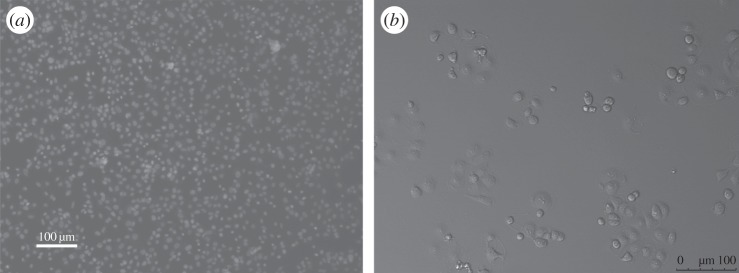
Images from two different *in vitro* cell biology experiments. (*a*) A distribution of 3T3 mouse fibroblast cells [19] which appears to be spatially uniform. The size of the image is 870×649 μm or 1976 pixels×1475 *pixels*, giving 0.44 μm *pixel*^−1^. (*b*) A distribution of MDA-MB-231 breast cancer cells [20] which appears to be highly clustered. The size of the image is 622×464 μm or 523 pixels×390 *pixels*, giving 1.19 μm *pixel*^−1^. Note that the objects in (*a*) correspond to cells which have been treated with a nucleus stain, whereas the objects in (*b*) correspond to cells without any nucleus stain.

This paper is organized in the following way. In §3, we outline our experimental and image processing techniques for generating and analysing images of certain *in vitro* cell biology experiments (e.g. figure 1). To test our methods, we use results from a random walk simulation model, which is also described in §3. In §4, we present results from our spectral technique for two different classes of images. First, we focus on synthetic and experimental data describing uniformly distributed objects. This allows us to demonstrate the key features and benefits of our proposed technique. Second, we focus on synthetic and experimental data describing aggregated objects. Our analysis of images of aggregated objects, in §4, includes a series of time-lapse images that illustrates the development of aggregates of cells in a population of breast cancer cells as a function of time during a growth-to-confluence assay. An interesting feature of this series of images is that the cells are initially distributed approximately uniformly, and we observe the formation of significant aggregation over time as the population grows. Applying our spectral technique to these images allows us to quantify various properties of the cellular growth process. Finally, in §5, we discuss the significance of our results and outline some options for future study.

## 3. Material and methods

### 3.1 Experimental methods

*3T3 culture.* Full details of the relevant cell culture technique are reported in Simpson *et al*. [1]. In brief, murine fibroblast 3T3 cells [19] were cultured in Dulbecco's modified Eagle medium (Invitrogen, Australia) supplemented with 5% fetal calf serum (FCS, Hyclone, New Zealand), 2 mM l-glutamine (Invitrogen) and 1% v/v penicillin/streptomycin (Invitrogen) in 5% *CO*_2_ at 37°C. Monolayers of 3T3 cells were cultured in T175 cm^2^ tissue culture flasks (Nunc, Thermo Scientific, Denmark). Cells were lifted just prior to confluence using 0.05% trypsin (Invitrogen) and viable cells were counted using a Trypan blue exclusion test and a haemocytometer. Before the 3T3 cell populations were imaged, a nuclear stain was applied to highlight the locations of the cell nuclei. The nucleus staining was applied by fixing the cells with 10% formalin. The cell membrane was made permeable using ice-cold 70% ethanol and the nucleus was stained with propidium iodide, 1 *mg* *ml*^−1^ (Invitrogen). This means that the objects highlighted in the images of the 3T3 populations correspond to the cell nuclei, which is significantly smaller than the total cell diameter.

*231 culture.* Full details of the relevant cell culture technique are reported in Simpson *et al*. [21]. In brief, MDA-MB-231 breast cancer cells [20] were cultured in Dulbecco's modified Eagle medium (Invitrogen) with 10% FCS (Hyclone) and 1% v/v penicillin/streptomycin (Invitrogen). Cells were cultured in T175 cm^2^ tissue culture flasks (Nunc, Thermo Scientific) kept in 5% CO_2_ at 37°C. Cells were lifted prior to confluence using 0.05% trypsin (Invitrogen) and viable cells were counted using a Trypan blue exclusion test and a haemocytometer. Unlike the 3T3 cells, the images of 231 cells were obtained without any nuclear staining. This means that the objects highlighted in the images of the 231 populations correspond to the entire cell.

To initiate each assay for both cell types, a cell suspension containing a known density of cells was created and carefully inserted into the wells of a 24-well tissue culture plate. This was to ensure that the initial distribution of cells was approximately uniform. For the 3T3 cells, images were taken using a Laborlux fluorescence microscope with a Nikon digital camera (DXM1200C). For the 231 cells, the assay was monitored in real time using a Leica AFLX 6000 widefield microscope. Experimental images, each covering an area of approximately 640×480 μ*m*, were recorded for both cell types. For the 3T3 cells we report these images at one time point only (e.g. figure 1*a*), whereas for the 231 cells we report a time series of such images (e.g. figure 1*b*). The images of the 231 cells were recorded every 200 min, over a total period of 2000 min.

### 3.2 Simulation methods

To generate snapshots of distributions of objects according to a controlled and specific mechanism, we use a discrete random walk model [22]. We note that this kind of model is sometimes referred to as an agent-based model [14]. In these simulations, we suppose that each individual ‘agent’ represents an individual cell among a population, and we aim to study and quantify different spatial arrangements of such agents using a binned pair-correlation function. In particular, we use this model to simulate two different kinds of distributions of objects:

(1) a spatially random distribution of objects, which we interpret as corresponding to an experimental image of a population of cells in which the dominant mechanism is random (undirected) cell motion without rapid proliferation or significant cell-to-cell adhesion [7], and
(2) a clustered distribution of objects, which corresponds to an experimental image where the dominant mechanism is rapid cell proliferation [5].

We generate images from our random walk model in a way that is consistent with how the experimental images are prepared. Simulations are performed on a relatively large 4*X*×4*X* unit square simulation lattice. The distribution of agents within a smaller *X*×*X* subregion of the simulation lattice is then analysed. The *X*×*X* subregion is located such that the centre of the *X*×*X* subregion coincides with the centre of the 4*X*×4*X* simulation lattice. This approach means that the boundaries of the images generated from our random walk model do not coincide with the boundaries of the underlying simulation lattice in exactly the same way that the boundaries of the experimental images do not coincide with the boundary of the tissue culture plate. Therefore, the distribution of agents in the images from our simulations extends well beyond the boundaries of the images in exactly the same way that the distribution of cells in the experimental images extends well beyond the boundaries of the experimental images. Our random walk simulations are performed with no-flux (reflecting) boundary conditions on all boundaries of the 4*X*×4*X* simulation lattice. This is consistent with the experimental set-up as cells cannot move across the physical boundary of the tissue culture plate.

**Figure 2.**
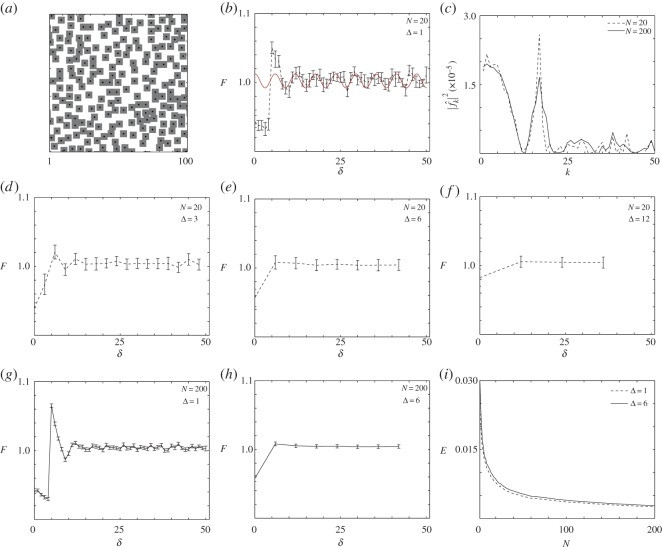
Simulation of a randomly placed distribution of agents, *s*=5, *ρ*=0.5, *X*= 100. (*a*) Typical realization. (*b*) Pair-correlation of agent locations for bandwidth Δ=1, *N*=20. The red curve is the rescaled filtered signal with wavelength λ=5.88 for the wavenumber *k*_1_=17. (*c*) Power spectrum with maximum wavenumber at *k*=17. The broken curve is for *N*=20 simulations. The solid curve is for *N*=200 simulations. (*d*–*f*) Pair-correlation for bandwidths Δ={3,6,12}, *N*=20. (*g*,*h*) Pair-correlation for bandwidths Δ={1,6}, *N*=200. (*i*) An estimate of the total error, given by equation (2.11), for Δ={1,6}. The error bars in (*b*,*d*–*h*) indicate the standard error, given by equation (2.10).

Simulations are initiated by placing agents on the simulation lattice uniformly at random. A sufficient number of agents is placed so that the initial density of agents in the *X*×*X* subregion is *ρ*(0)=*ns*^2^/*X*^2^, where *n* is the number of square agents and *s*^2^ is the area of each agent. Our model is an exclusion process [23,24], and so we take care to ensure that agents are placed on the lattice such that no agents overlap. As this initial condition is placed at random, we use images of this initial placement to represent a random distribution of objects (e.g. figure 2*a*) which is free from spatial correlations. Alternatively, to generate a clustered distribution of objects, we take the random initial distribution of agents and allow the system to evolve by implementing an unbiased proliferation mechanism. We discretize time using constant time steps, each of duration *τ*, and implement a random sequential update method [25]. In each time step, *n* agents are selected, at random, with replacement, and given the opportunity to proliferate (or reproduce) with probability *R*∈[0,1]. During any proliferation event, an attempt will be made to place a daughter agent onto the lattice, adjacent to the mother agent. The direction in which the daughter agent is placed is chosen, at random, from one of the four possible directions imposed by the square lattice structure. As our random walk model is an exclusion process [23,24], any attempted proliferation event that would place a daughter agent on a lattice site which is already occupied is aborted. Simulations are terminated at some time *T*, producing highly clustered spatial distributions of agents (e.g. figure 4*a*).

To analyse the resulting spatial distributions of agents produced by our random walk model, we identify the cental locations of each agent on the lattice (e.g. indicated by the black markers in figures 2*a* and 4*a*). To generate averaged information from our model, we always consider generating a suite of *N* identically prepared simulations using the same random walk mechanism.

### 3.3 Image analysis methods

We analyse the experimental cell biology images by identifying the location of individual objects within the experimental images using customized software written with MATLAB's Image Processing Toolbox [5,14,26]. Owing to the differences in the experimental procedure, the objects identified in the images of the 3T3 populations correspond to the cell nuclei, whereas the objects identified in the images of the 231 populations correspond to the entire cells.

**Figure 3.**
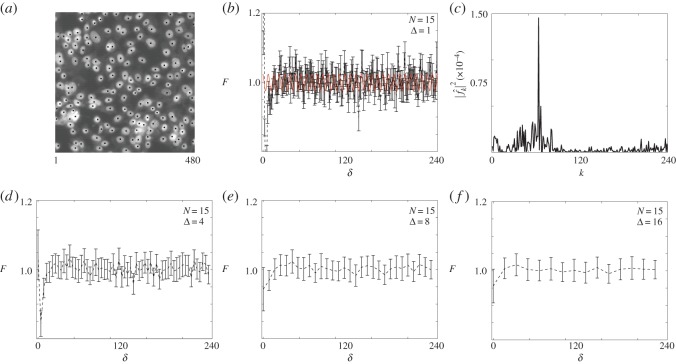
Distribution of highly motile 3T3 fibroblast cells, *X*=480. The average number of cells per image is $\bar{n}=147.07$, with standard deviation *σ*=17.87 from *N*=15 samples. (*a*) Cell locations superimposed (black markers) on a sample image. (*b*) Pair-correlation of cell locations for bandwidth Δ=1. The red curve is the rescaled filtered signal with wavelength λ=7.5 for the wavenumber *k*_1_=64. (*c*) Power spectrum with a maximum wavenumber at *k*=64. (*d*–*f*) Pair-correlation for bandwidths Δ={4,8,16}. The error bars in (*b*,*d*–*f*) indicate the standard error, given by equation (2.10).

Non-overlapping square sub-images, each of size *X*×*X* pixels, with X∈2N, are extracted from the experimental images (e.g. figure 1). Each sub-image is processed by manually determining the location of each object in the image, and denoting the location of each object by superimposing markers on the sub-image (e.g. figures 3*a* and 5*a*,*d*). We treat each processed sub-image as a single sample in a total of *N* independent, identically prepared images. For the images of the 3T3 populations, we analysed three distinct sub-images, with *X*=480 pixels, from each of five original larger images (e.g. figure 1*a*), giving a total of *N*=15 samples. For the images of the 231 populations, one sub-image with *X*=390 pixels was extracted from 17 distinct images (e.g. figure 1*b*), giving a total of *N*=17 samples. We note that the 3T3 images all correspond to one particular time point in the original experiment, whereas the 231 images correspond to a time series of images taken at evenly spaced time intervals so that we could gather information about how the total numbers of 231 cells and their spatial arrangement changed with time. More details about this time series is given in §4.

Before we present any results from our image analysis or spectral methods, we first discuss the choice of spatial units. As we analyse the experimental images using MATLAB's Image Processing Toolbox [14,5], which discretizes all images into pixels, we present results using pixels as the relevant length scale. Although it is possible to convert any of these length measurements, or inferred length scales, into a physical measure (where the relevant units are micrometres), we prefer to present all results in terms of pixels as this is a natural unit of length associated with automatic image processing tools. For completeness, we note that the conversion factor required to convert between pixels and micrometres for the images of 3T3 and 231 cells are given by

3.1 1 pixel=0.44 μ m

and

3.2 1 pixel=1.19 μ m,

respectively. The difference in conversion factor for the two different types of images is due to the fact that the images of the 3T3 populations and the 231 populations were obtained using different microscopes.

### 3.4 Spatial statistics and spectral methods

To quantify the spatial pattering of cell locations in the processed images, we calculate an average periodic pair-correlation function [8,12–14,27]. The pair-correlation is a scaled count of the (periodic) pair-distances, in either the *x* or *y* Cartesian direction that accounts for all possible combinations of paired cell locations in a given image. Following our previous work [14,8], we begin by formulating the pair-correlation function in the *x*-direction and will later comment on how the methodology can be applied to calculating the pair-correlation function in the *y*-direction. As we have explained in §3.1 and 3.2, the experimental and simulation images that we analyse show snapshots of a subregion containing agents and cells. These subregions are contained within a much larger region and hence the boundary of the simulation and experimental images presented in this work are not physical boundaries and the distribution of agents and cells extends well beyond the boundary of these images. Therefore, it is more natural for us to implement a periodic pair-correlation function [8] than a non-periodic pair-correlation function [14].

Consider the *i*th sample image consisting of *X*×*X* pixels with a total of *n*^*i*^ cell locations identified at some of the integer lattice sites *x*=1,…,*X* and *y*=1,…,*X*, where X∈2N. For any two distinct cell locations in the *x*-direction, *x*=*x*_1_ and *x*=*x*_2_, we define the periodic pair-distance as $\delta =\min\{|x_{1}-x_{2}|,X-|x_{1}-x_{2}|\}$. The frequency, or counts, of the pair-distances between all possible combinations of paired cell locations, in the *i*th image, is denoted by $c_{x}^{i}(\delta )$, for *δ*=0,…,*X*/2. The counts of the pair-distances are scaled, or normalized, with respect to the expected counts of pair-distances on an integer lattice populated at random [10,11,28,29], giving the periodic pair-correlation function in the *x*-direction of the *i*th sample image as

3.3 $$P_{x}^{i}(\delta )=\frac{c_{x}^{i}(\delta )d(\delta )(X^{2}-1)}{Xn^{i}(n^{i}-1)},\;\text{for}\;\delta =0,\ldots ,\frac{X}{2},$$

where *d*(*δ*)=2 for *δ*∈{0,*X*/2}, and *d*(*δ*)=1 for *δ*∈{1,…,*X*/2−1}. We note that the pair-correlation function in the *y*-direction, $P_{y}^{i}(\delta )$, can be calculated in a similar way, simply by considering counts of pairs of objects in the *y*-direction, $c_{y}^{i}(\delta )$. For spatial patterning with no directional bias in either Cartesian direction, we can define an isotropic periodic pair-correlation function

3.4 $$f_{i}(\delta )=\frac{1}{2}[P_{x}^{i}(\delta )+P_{y}^{i}(\delta )],\;\text{for}\;\delta =0,\ldots ,\frac{X}{2}.$$

The average isotropic pair-correlation function is then

3.5 $$f(\delta )=\frac{1}{N}\sum_{i=1}^{N}f_{i}(\delta ),\;\text{for}\;\delta =0,\ldots ,\frac{X}{2},$$

for which the standard error is given by

3.6a $$\sigma (\delta )=\sqrt{\frac{1}{N(N-1)}\sum_{i=1}^{N}{\left[f_{i}-f(\delta )\right]}^{2}},\;\text{for}\;\delta =0,\ldots ,\frac{X}{2}.$$

A measure of the total error is

3.7a $$E(\sigma ,N)=\frac{1}{X/2+1}\sum_{\delta =0}^{X/2}\sigma (\delta ).$$

Given the isotropic pair-correlation data, described by equation (2.4), we can construct a box-kernel smoothed estimate, with bandwidth Δ∈N, given by

3.8 $$F_{i}(j)=\frac{1}{\Delta }\sum_{\delta =j\Delta }^{(j+1)\Delta -1}f_{i}(\delta ),\;\text{for}\;j=0,\ldots ,\left\lfloor \frac{X}{2\Delta }+\frac{1}{\Delta }\right\rfloor -1.$$

The mean of this estimate is given by

3.9 $$F(j)=\frac{1}{N}\sum_{i=1}^{N}F_{i}(j)=\frac{1}{\Delta }\sum_{\delta =j\Delta }^{(j+1)\Delta -1}f(\delta ),\;\text{for}\;j=0,\ldots ,\left\lfloor \frac{X}{2\Delta }+\frac{1}{\Delta }\right\rfloor -1,$$

and the standard error is given by

3.6 $$\nu (j)=\sqrt{\frac{1}{N(N-1)}\sum_{i=1}^{N}{\left[F_{i}(j)-F(j)\right]}^{2}},\;\text{for}\;j=0,\ldots ,\left\lfloor \frac{X}{2\Delta }+\frac{1}{\Delta }\right\rfloor -1.$$

An estimate of the total error is

3.7 $$E(\nu ,N)=\frac{1}{\left\lfloor X/2\Delta +1/\Delta \right\rfloor }\sum_{j=0}^{\lfloor X/2\Delta +1/\Delta \rfloor -1}\nu (j).$$

We note that the expressions for the binned data, given by equations (2.9) and (2.11), are equivalent to the expressions for the unbinned data, given by equations (2.5) and (2.7), respectively, when Δ=1. As we indicated in §2, the main focus of this work is to present an objective method to assist in identifying an appropriate bandwidth, Δ.

In this work, we propose that, for a given dataset, the bandwidth, Δ, can be chosen by examining the pair-correlation signal, equation (2.5), in spectral space. Before transforming the signal into spectral space, we first modify *f*(*δ*) to obtain a 2*π* periodic symmetric function. This is achieved by first letting $\tilde{f}(\tilde{\delta })=\tilde{f}(-\tilde{\delta })=f(\delta )$, with $\tilde{\delta }=2\delta \pi /X$, and then taking the discrete Fourier transform [30]

3.12 $${\hat{f}}_{k}=\frac{1}{Xc_{k}}\sum_{l=0}^{X-1}\tilde{f}({\tilde{\delta }}_{l})\;e^{-ik{\tilde{\delta }}_{l}},\;k=0,\pm 1,\ldots ,\pm \frac{X}{2}.$$

Here, ${\tilde{\delta }}_{l}=-\pi +2\pi l/X$, *l*=0,…,*X*−1; *c*_*k*_=2 for *k*=±*X*/2, and *c*_*k*_=1 for *k*≠±*X*/2.

Examining the power spectrum, $|{\hat{f}}_{k}{|}^{2}$, provides a systematic way to identify dominant modes, or wavenumbers, of the modified pair-correlation function $\tilde{f}$, with wavelengths $\tilde{\lambda }=2\pi /k$. The corresponding wavelengths for the pair-correlation function *f*, given by equation (2.5), are $\lambda =\tilde{\lambda }X/2\pi =X/k$.

In this work, we identify two dominant modes and filter the pair-correlation signal by considering the subset of wavenumbers

3.13 $$K=\{k|k=0,|k|=k_{1}\}.$$

The filtered (continuous) pair-correlation signal is given by taking the (re-scaled) inverse discrete Fourier transform

3.14 $$f_{K}(\hat{\delta })=\sum_{k\in K}{\hat{f}}_{k}\;e^{2\pi \text{i}k\hat{\delta }/X},\;0\leq \hat{\delta }\leq \frac{X}{2},\;\text{for}\;\hat{\delta }\in R.$$

The wavenumber *k*=0 corresponds to a constant value in physical space, with the *k*=*k*_1_ mode being related to a cosine wave, due to symmetry of the modified pair-correlation function. The wavelength, λ, of the cosine wave provides a characteristic length scale in the spatial analysis, which we propose ought to be taken as the bandwidth, Δ, in the pair-correlation signal defined by equation (2.9). We will now present results, for both synthetic and experimental data, which support this claim.

## 4. Results

### 4.1 Random synthetic data

We begin the spatial analysis by considering a simulation of randomly placed objects, which, as we noted in §3, could correspond to an image of a population of cells in which the dominant mechanism is undirected, random motility. Such an image is shown in figure 2*a*. This image highlights the importance of choosing an appropriate bandwidth to interpret the pair-correlation signal as the unbinned (Δ=1) pair-correlation signal in figure 2*b* implies that:

(1) the domain is segregated at length scales *δ*<5, as *F*<1 over these distances,
(2) the domain is aggregated within the interval 6<*δ*<10, as *F*>1 over these distances, and
(3) that the spatial patterning has no structure for *δ*>10, as *F* fluctuates around unity over these distances.

This example highlights the usefulness of analysing synthetic simulation data, as, in this case, we know in advance that the spatial distribution of agents in figure 2*a* is completely random, yet the pair-correlation function obtained using an inappropriate bandwidth could lead us to draw incorrect conclusions about the spatial distribution.

The power spectrum, $|{\hat{f}}_{k}{|}^{2}$, shown in figure 2*c*, provides information to assist our choice of bandwidth for this randomly placed synthetic data. In particular, the power spectrum contains a maxima at *k*=17, which corresponds to a re-scaled wavelength of λ=5.88. The filtered signal is shown as the sinusoidal curve superimposed in figure 2*b*. These results indicate that a potential bandwidth for the pair-correlation signal is the nearest integer value of the wavelength, Δ=[λ]=6, and the appropriately binned kernel density estimate of pair-correlation data, shown in figure 2*e*, provides a more accurate summary of the expected features of this spatial pattern. In particular, the appropriately binned pair-correlation signal implies that the pattern is segregated at length scales *δ*<6, and this feature can be attributed to the exclusion mechanism which means that individual objects cannot be placed any closer than the length scale of the individual objects, *s*=5. For the remaining length scales in the domain, the appropriately binned pair-correlation function fluctuates about unity, which correctly indicates that there is no spatial structure at these length scales. For completeness, we also show, in figure 2*d*,*f*, two additional signals where the data has been binned both below (Δ=3, figure 2*d*) and above (Δ=12, figure 2*f*) the appropriate value determined using the spectral technique. Comparing results in figure 3*d*–*f* confirms that choosing a smaller value of Δ leads to an overly noisy pair-correlation signal, which may lead us to draw incorrect conclusions about the spatial patterning present in the image. Alternatively, choosing Δ too large leads to an overly smoothed signal which may mask the relevant features.

Overall, in comparing our four different pair-correlation signals in figure 2*b*,*d*–*f*, we see that when we choose the appropriate value of Δ there are no spurious spikes in the pair-correlation signal above unity in figure 2*e*, whereas the inappropriately binned results in figure 2*b*,*d* contain spurious spikes, above unity, which we know are misleading as the objects in question have been placed at random. Furthermore, comparing our results in figure 2*e*–*f* indicates that choosing an inappropriately large bandwidth, Δ>6, incorrectly indicates that the domain is segregated at length scales of approximately *δ*<10, which we also know is misleading as all objects in the image have a length scale of *s*=5. This means that there is no segregation at distances larger than *s*=5.

In summary, our analysis of the synthetic randomly placed objects in figure 2*a* allows us to illustrate the potential for the misinterpretation of pair-correlation signals. The advantage of using this synthetic data is that we know that the objects are placed at random, and furthermore we know the size of all objects before any analysis is attempted. These features mean that the pair-correlation signal should be less than unity for length scales smaller than the length scale of the objects and that the pair-correlation signal should fluctuate around unity for larger length scales. Results in figure 2*b*,*d* show that choosing Δ to be too small leads to a misleading result, which could indicate that the objects are aggregated, whereas the results in figure 2*f* also lead to a misleading result since choosing Δ to be too large indicates that the size of the object is too small. Conversely, our choice of Δ=6, which was found by analysing the power spectrum of the pair-correlation signal, provides an appropriate pair-correlation signal in figure 2*e*. This reflects the expected properties of this synthetic data.

We also provide additional data about the role of the number of samples, *N*, in figure 2. The pair-correlation signals in figure 2*g*–*h* correspond to increasing the number of samples to *N*=200 for Δ=1 and Δ=6, respectively. Comparing these pair-correlation signals with the results in figure 2*b*,*e* with *N*=20 confirms that we observe similar quantitative trends regardless of whether we consider *N*=20 or *N*=200 identically prepared realizations of the discrete model. Most importantly, when we repeat our spectral analysis with *N*=200, we see that the maximum wavenumber in figure 2*c* is the same regardless whether we consider *N*=20 or *N*=200. The results in figure 2*i* give an estimate of the error (given by equation (2.11)) as a function of the sample size, confirming that the fluctuations in the pair-correlation signal decay with *N*, as expected.

Given that most realistic distributions of objects in images from cell biology experiments are not placed at random, and we do not know the precise size of the objects *a priori*, we will now analyse more detailed images to explore how our spectral analysis performs under more realistic conditions.

### 4.2 Random experimental data

The image in figure 3*a* shows a distribution of 3T3 cells, which are known:

(1) to undergo proliferation at a rate which is small compared with the rate of motility [1,21] and
(2) to be relatively unaffected by cell-to-cell adhesion [31,32].

Under these circumstances, we expect the distribution of 3T3 cells in figure 3*a* to be approximately spatially uniform; however, unlike the distribution of synthetic objects in figure 2*a*, in an experimental image we may not know the precise size of the objects *a priori*. To provide some preliminary insight into the spatial pattern in figure 3*a,* we present the pair-correlation signal in figure 3*b* with Δ=1, which gives a complicated signal containing,

(1) a region (*δ*<5) containing a large spike, above unity, indicating aggregation,
(2) a region (5<*δ*<25) which indicates that the objects are segregated, and
(3) a region (*δ*>25) in which the signal fluctuates about unity.

To obtain further insight into the reliability of the information contained in this initial signal, we compute the power spectrum and the results in figure 3*c* indicate a dominant wavenumber *k*=64, corresponding to a wavelength of λ=7.5. Results in figure 3*e* show the pair-correlation data binned at Δ=8, as indicated by our spectral analysis, and the results in figure 3*d*,*f* allow us to compare the pair-correlation data binned using an appropriate choice of Δ relative to two other sets of pair-correlation data using smaller (Δ=4) and larger (Δ=16) bandwidths, respectively. The results in figure 3*e* imply that the objects are distributed close to uniform for *δ*>25 and that the objects are segregated over distances *δ*<25. Using equation (2.1), this implies that the length scale of the objects is approximately 11 μ*m*. This result appears to be reasonable since the spectral analysis identifies the length scale of the cell nucleus, as shown in figure 3*a*, which is smaller than the total cell diameter [33]. Of course, this estimate is an approximation as we expect that our results are affected by the fact that there is some degree of variability in the shape and size of the cells among the population. Comparing the results in figure 3*d*–*f* confirms that choosing Δ too small can lead us to make inappropriate conclusions about the spatial patterning, as the results in figure 3*d* imply that there is very short-scale aggregation present. Conversely, the pair-correlation signal in figure 3*f* confirms that choosing Δ too large could lead us to underestimate the size of the objects in the image.

In this section, we have analysed one set of images showing a distribution of 3T3 cells at one time point during an experiment. Of course, had we calculated an appropriately binned pair-correlation signal for a number of different time points during the assay, our approach could provide insight into any temporal changes in the spatial patterning. In §4.4, we provide such a time series and analyse the corresponding time series of pair-correlation signals.

### 4.3 Clustered synthetic data

**Figure 4.**
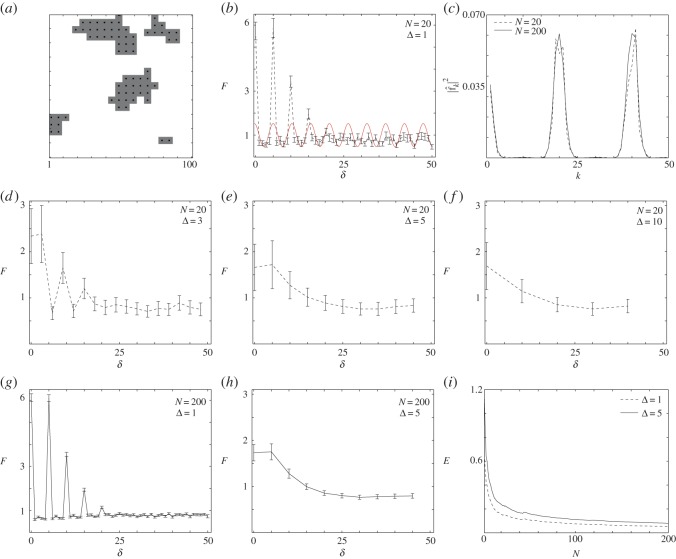
Synthetic distribution of highly proliferative agents, *s*=5, t=5 min, *τ*=1 min, *ρ*(0)=0.01, *R*=1, *X*=100. (*a*) Typical realization. (*b*) Pair-correlation of agent locations for bandwidth Δ=1, and *N*=20. The red curve is the rescaled filtered signal with wavelength λ=5.26 for the wavenumber *k*_1_=19. (*c*) Power spectrum with maxima wavenumbers at *k*=19 and *k*=41. The broken curve is for *N*=20 identically prepared simulations. The solid curve is for *N*=200 identically prepared simulations. (*d*–*f*) Pair-correlation for bandwidths Δ={3,5,10}, *N*=20. (*g*,*h*) Pair-correlation for bandwidths Δ={1,5}, *N*=200. (*i*) An estimate of the total error, given by equation (2.11), for Δ={1,5}. The error bars in (*b*,*d*–*h*) indicate the standard error, given by equation (2.10).

Having demonstrated the effectiveness of the spectral method at selecting an appropriate bandwidth in both the synthetic and experimental images containing randomly distributed objects, we now analyse additional synthetic data. This has been produced by the random walk algorithm and contains clustered objects. The image showing the clustered synthetic data, figure 4*a*, is generated by placing a small number of agents, at random, on the lattice at *t*=0 and allowing the unbiased proliferation mechanism to evolve for a short time until we observe the formation of visually well-defined aggregates. The pair-correlation data in figure 4*b*, with Δ=1, contains four large spikes above unity for *δ*<16, with the signal fluctuating around a constant value, less than unity, when *δ*>16. Unlike the randomly placed synthetic data in figure 2*a*, in this case we expect there to be some degree of aggregation owing to the way in which the proliferation mechanism gives rise to clusters of agents [14]. Therefore, while we anticipate that we will see some region of the pair-correlation function above unity, we are unsure, at this preliminary stage, whether the initial signal in figure 4*b* provides us with an accurate depiction of the real spatial pattern present in the image. To provide further insight, we present the power spectrum in figure 4*c* which suggests that we have two dominant wavenumbers, *k*=19 and *k*=41, corresponding to λ=5.26 and λ=2.43, respectively. This result reduces the candidate wavenumbers from 50 (*X*/2=50) to just two, which we can use to construct the binned pair-correlation signal. In this case, we expect that we will be able to discriminate between these two potential choices of bandwidths since the appropriately binned pair-correlation signal should allow us to characterize the length scale of the objects in the image, which in this case we know to be *s*=5 as we are dealing with synthetic data.

The pair-correlation signal in figure 4*e*, binned with Δ=5, indicates that:

(1) the spatial patterning is aggregated at length scales of *δ*<20,
(2) the signal is approximately constant, less than unity, when *δ*>20, implying that the clusters are distributed randomly, and
(3) the pair-correlation signal approximately intersects with unity at *δ*≈20, which is an indication of the aggregate size [14].

Comparing the pair-correlation signals in figure 4*d*–*f* confirms, as before, that choosing Δ too small can lead us to make inappropriate conclusions about the size of the objects present in the image. By contrast, choosing Δ too large can lead to an overly smooth signal which can hide some of the detail.

We also provide additional data about the role of the number of samples, *N*, in figure 4. The pair-correlation signals in figure 4*g*–*h* correspond to *N*=200 for Δ=1 and Δ=5, respectively. Comparing these signals with the results in figure 4*b*,*e* confirms that we observe similar quantitative trends regardless of whether we consider *N*=20 or *N*=200. Repeating our spectral analysis with *N*=200, we see that the dominant wavenumbers in figure 4*c* are similar for *N*=20 and *N*=200. This result is of great practical interest as it is experimentally feasible to deal with *N*=20 identically prepared images, whereas generating *N*=200 identically prepared experimental images may be sufficiently time consuming and expensive that this number is impractical. Results in figure 4*i* give an estimate of the error (given by equation (2.11)) as a function of sample size, confirming that the fluctuations in the pair-correlation signal decay with *N*, as expected.

Unlike the synthetically generated image in figure 4*a*, when we analyse real experimental images of clustered cells, we may not have any precise *a priori* estimate of the size of the objects which make up the aggregates. It would be more usual for us to have an estimate of the object size based on our basic knowledge of cell biology, and we anticipate that this may be sufficient for us to discriminate between potential choices of bandwidth for those situations, analogous to the results in figure 4*c*, where the power spectrum contains two or more dominant wavenumbers. To explore how our approach deals with more realistic images, we now focus on a set of experimental images which contain clustered aggregates of cells.

### 4.4 Clustered experimental data

**Figure 5.**
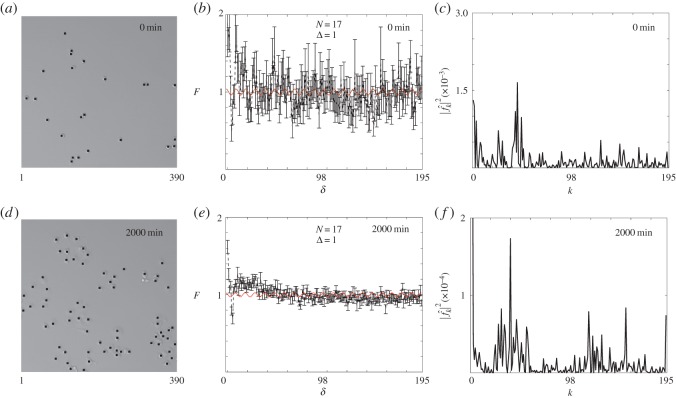
Experimental images of highly proliferative 231 breast cancer cells, *X*=390 and *N*=17. Images in (*a*,*d*) show the location of cells superimposed (black markers) on sample images at time 0 and 2000 min. (*b*,*e*) Pair-correlation of cell locations at time 0 and 2000 min for bandwidth Δ=1. The red curves are the rescaled filtered signals with wavelength λ=9.75 for the wavenumber *k*_1_=40. (*c*,*f*) Power spectra at time 0 and 2000 min with maximum wavenumbers at *k*=45 and *k*=40, respectively. The error bars in (*b*,*e*) indicate the standard error, given by equation (2.10).

Here, we apply our spectral method to images of clustered 231 cells in figure 5*a*,*d*. These images summarize a growth-to-confluence assay in which a relatively small number of cells is initially distributed, approximately uniformly (figure 5*a*), and then after some time the relatively immotile cells proliferate to form visually distinct clusters (figure 5*d*) [1]. Unlike the 3T3 cells, which move fast relative to their rate of proliferation [1], the 231 cells move relatively slowly compared with the rate of proliferation, and the net result of this is the formation of visually discernible clusters of 231 cells [1]. To explore how our spectral method can provide information about these images, we show the pair-correlation function, with Δ=1, in figure 5*b* for the distribution of cells at t=0 min and note that it is very difficult to meaningfully interpret this signal as it is overly noisy. As this pair-correlation function has been constructed using images corresponding to the initial part of the experiment, where the number of cells per image is relatively small, we might anticipate that the fluctuations in the signal will be partly due to the small number of cells in the image. Despite the potential difficulties with the small numbers of cells, the power spectrum in figure 5*c* suggests a candidate wavenumber of *k*=45, corresponding to a wavelength of λ=8.67. The snapshot of the growth process in figure 5*d* shows that after 2000 min, the number of cells have increased dramatically, and we anticipate that we will have a smoother pair-correlation signal as the number of pairs of objects increases as $O(n^{2})$, where *n* is the number of cells per image. The pair-correlation signal with Δ=1, shown in figure 5*e*, is far less noisy and the power spectrum in figure 5*f* suggests a candidate wavenumber at *k*=40, corresponding to a wavelength of λ=9.75. From this preliminary information, we anticipate that constructing the pair-correlation signal using Δ=10 would be reasonable.

**Figure 6.**
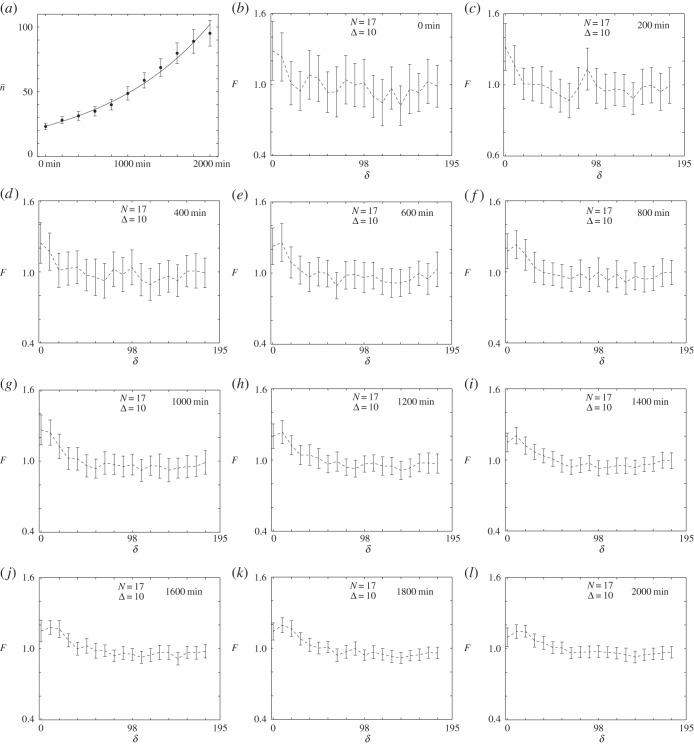
Spatial analysis of a set of time-series images depicting a growth-to-confluence experiment conducted with highly proliferative 231 breast cancer cells, *X*=390 and *N*=17. Results in (*a*) show the average cell numbers $\bar{n}(t)$ (markers), the error bars indicate the standard error of the mean. An exponential growth curve, n(t)=n(0)exp⁡[αt], fitted to the data points, with $\bar{n}(0)=23.33$, *α*=0.0443 and coefficient of determination *R*^2^=0.99, is superimposed on the data. Results in (*b*–*l*) show the time evolution of pair-correlation constructed using Δ=10. The time of the experiment is given in each sub-figure and the error bars in (*b*–*l*) indicate the standard error, given by equation (2.10).

The images in figure 5*a*,*d* illustrate the time evolution of the 231 growth-to-confluence process at t=0 min and t=2000 min, respectively. A summary of the average numbers of cells present per image for the entire period, 0≤t≤2000 min, is given in figure 6*a*, confirming that the total number of cells present per images increases by approximately a factor of four over this time interval. We now analyse the spatial patterning present in these images, every 200 min, by constructing a time series of average pair-correlation signals with Δ=10, as shown in figure 6*b*–*l*. Comparing the shape and details of this series of the pair-correlation signals reveals several pieces of information about the growth process. Three conclusions can be drawn from the time series in figure 6*b*–*l*:

(1) the pair-correlation signal contains less fluctuations as time increases, and we attribute this to the fact that the number of cells per image increases by a factor of four during the 2000 min growth process, which means that the number of pairs of cells increases dramatically during the growth-to-confluence assay,
(2)  the pair-correlation signals corresponding to the later part of the growth-to-confluence assay (e.g. t>400 min) confirm the presence of short-range correlations as there is a region, approximately *δ*<50, over which the distribution is aggregated, whereas for all distances larger than 50 the pair-correlation signal is approximately constant, indicating a lack of spatial structure at these larger length scales, and
(3) comparing the point at which each pair-correlation signal intersects with *F*=1 at early time (e.g. *t*=400 *mins*) and later time (e.g. *t*=2000 *mins*) confirms that the average size of the clusters increases with time, from approximately 50 to approximately 70, during this 1600 min time interval.

## 5. Discussion and conclusion

Observations of cell clustering are relatively common and can have important implications, both *in vivo* and *in vitro*. For example, melanoma is responsible for most deaths from skin cancer, and early detection is essential for successful treatment. Many of the physiological properties associated with melanoma (e.g. asymmetry, irregular border, colour irregularity [3,4]) could be associated with cell clustering effects [5]. In an *in vitro* context, the presence or absence of clustering in two-dimensional cell culture assays is known to provide valuable information about the relevant mechanisms (e.g. rapid proliferation, strong cell-to-cell adhesion) controlling the collective behaviour of the cell populations [8]. Given the importance of cell clustering, it is essential to develop reliable computational tools which can be used to provide quantitative insight into certain properties of clustered distributions, such as quantifying the average size of the clusters of objects and quantifying the average size of the objects in the image.

Our recent work has developed a pair-correlation function which can be used to analyse certain properties of images depicting populations of potentially clustered objects [10–14]. In particular, our pair-correlation function can be used to distinguish between spatial patterns which involve uniformly distributed objects, aggregated objects and segregated objects, as well as providing estimates of the average size of objects and the average size of aggregates of objects within the images. One aspect of implementing our pair-correlation function, which we did not explore previously, was the choice of bandwidth used when constructing kernel density estimates of the pair-correlation function. We note that previous studies have indicated that the choice of bandwidth can have a major impact on the pair-correlation signal, and previous investigations have suggested heuristic methods, including trial-and-error, to determine the appropriate bandwidth [13,27].

Our present results confirm that choosing an inappropriately large bandwidth results in the pair-correlation function containing insufficient information as the length scales of patterning are not detected. Alternatively, choosing an overly small bandwidth leads to the pair-correlation function being dominated by fluctuations which means that it is difficult to identify meaningful features in the pair-correlation signal. A key feature of our study is that we analyse synthetically generated images in which we know, in advance, whether the pattern is uniform or clustered, and what the size of the objects in the image are. By analysing such synthetically generated data, we are able to test the effectiveness of our proposed strategy for determining an appropriate bandwidth by analysing the power spectrum of the discrete Fourier transform of the unbinned pair-correlation function. By analysing both clustered and uniform synthetic data, we show that the power spectrum is dominated by just one or two dominant modes which provide us with a candidate bandwidth. For both uniform and clustered data, we show that when we construct the binned pair-correlation kernel density estimate using the candidate bandwidth suggested by the new spectral technique, we recover the expected properties of the spatial distribution. Our method is successful because the discrete Fourier transform of the pair-correlation function acts to filter the fluctuations in the pair-correlation signal in spectral space, allowing us to identify wavenumbers corresponding to physically relevant length scales.

To demonstrate how our choice of bandwidth applies to experimental images we apply the technique to two different sets of images: first, we analyse a series of images of a population of 3T3 fibroblast cells that are thought to be distributed randomly; and second, we analyse a time series of images from a growth-to-confluence assay using 231 breast cancer cells where the formation of aggregates of cells is visually obvious during the growth process. For the images of the 3T3 cells, our spectral technique confirms that the cells are uniformly distributed at sufficiently large length scales as well as indicating that the size of the objects in the images, which correspond to the 3T3 cell nuclei, are, on average, approximately 11 μ*m*. For the images of the 231 breast cancer cells, our spectral technique confirms the presence of short-range aggregation in the time series of images summarizing the growth-to-confluence assay. In particular, our results are consistent with previous investigations which indicate that the developing monolayer of 231 cells forms through a mechanism whereby the initially isolated and relatively immotile cells proliferate to form isolate aggregates which grow in size with time [1].

We conclude by acknowledging some of the limitations of the present study and outlining some potential avenues for future investigation. All of the images analysed in this study correspond to relatively simple experimental and modelling scenarios, whereby we treat the population of objects as being composed of a single subpopulation. While such mono-culture assays provide important insight into various biological processes, co-culture assays study a population of cells composed of multiple cell types (or subpopulations of cells). Such co-culture assays can provide more realistic insights into many biological processes including tissue repair, tissue regeneration and malignant spreading [34,35]. An interesting and relevant extension of our present work is to consider constructing pair-correlation functions for co-culture assays involving two or more distinct subpopulations of cells in which we make a distinction between pairs of cells from the same subpopulation and pairs of cells from distinct subpopulations. Under these more complicated conditions, we anticipate that techniques for estimating an appropriate choice of the bandwidth will be more delicate, as it is possible that such co-culture systems could involve subpopulations which may interact in more complicated ways to produce far more detailed spatial patterning. A further relevant extension of this work would be to make an attempt at understanding the role of variability in shape and size of the objects in the spatial pattern. Visual inspection of the experimental image in figure 3*a* indicates that, as we expect, biological cells are variable in shape and size, and it would be interesting to apply the techniques developed in this work to more detailed images where the variability in shape and size of the objects is more pronounced so that we can gain an understanding of how much information can be reliably obtained about more complicated spatial patterns.

## Supplementary Material

File 1: Data for 3T3 images (data3T3.mat)

## Supplementary Material

File 2: Data for 231 images (data231.mat)

## Data accessibility

All experimental data required to repeat the analysis in this article is included in the electronic supplementary material. The location data corresponding to the 231 and 3T3 images are stored separately in two MATLAB files, data231.mat and data3T3.mat, respectively. Each file contains a four-dimensional array with binary entries: an entry of one indicates the location of a cell. The first and second dimensions in the array correspond to the *x* and *y* Cartesian coordinates of the objects in the images, the third dimension corresponds to the time point and the fourth dimension indicates the sample number.
